# Selection of the Optimal Spectral Resolution for the Cadmium-Lead Cross Contamination Diagnosing Based on the Hyperspectral Reflectance of Rice Canopy

**DOI:** 10.3390/s19183889

**Published:** 2019-09-09

**Authors:** Shuangyin Zhang, Ying Zhu, Mi Wang, Teng Fei

**Affiliations:** 1 State Key Laboratory of Information Engineering in Surveying, Mapping and Remote Sensing, Wuhan University, Wuhan 430079, China; shyzh@whu.edu.cn; 2 School of Resource and Environmental Sciences, Wuhan University, Wuhan 430079, China; feiteng@whu.edu.cn

**Keywords:** heavy metal pollution diagnosis, hyperspectral remote sensing, optimal spectral resolution selection, cross contamination

## Abstract

This paper proposed an optimal spectral resolution for diagnosing cadmium-lead (Cd-Pb) cross contamination with different pollution levels based on the hyperspectral reflectance of rice canopy. Feature bands were sequentially selected by two-way analysis of variance (ANOVA2) and random forests from the high-dimensional hyperspectral data after preprocessing. Then Support Vector Machine (SVM) was applied to diagnose the pollution levels using different feature bands combination with different spectral resolutions and cross validation was conducted to evaluate the distinguishing accuracies. Finally, the optimal spectral resolution could be determined by comparing the diagnosing accuracies of the optimal feature bands combination in each spectral resolution. In the experiments, the hyperspectral reflectance data of rice canopy with ten different spectral resolutions was captured, covering 16 pretreatments of Cd and Pb pollution. The experimental results showed the optimal spectral resolution was 9 nm with the highest average accuracy of 0.71 and relatively standard deviation of 0.07 for diagnosing the categories and levels of Cd-Pb cross contamination. The useful exploration provided an evidence for optimal spectral resolution selection to reduce the cost of heavy metal pollution diagnose.

## 1. Introduction

Heavy metal pollution is seriously jeopardizing food security, and this hazard is continuing to intensify due to the increasing byproducts of frequent anthropogenic activities, such as industrial pollutants, wastewater, mining wastes, and pesticides [1,2]. Traditional methods for heavy metal diagnosis rely on wet chemistry analysis of a collection of soil samples, which has low-efficiency and is time-consuming [3,4], so high-efficiency and time-saving visible-near infrared reflectance spectroscopy (VNIRS) has become an alternative technology for diagnosing heavy metal pollutions. 

Some investigations have shown the feasibility of VNIRS based methods to diagnose the polluted or stressed categories the plants suffer, including pest stress, salinity stress, water stress, and heavy metal pollution [5,6,7,8,9,10,11,12,13]. These studies explored its diagnostic ability on the basis of soil samples hyperspectral datasets measured by VNIRS in the laboratory. For non-heavy metal diagnosis, Moshou et al. and Huang et al. [5,14] explored the feasibility of using the spectral reflectance of wheat to detect pest stress. Wang et al. [6] explored the possibility of interpreting water stress from plants using VNIRS. Tilley et al. and Rud et al. [7,15] investigated the feasibility of distinguishing salinity stress from plants using VNIRS. For heavy metals pollution diagnosis, some researchers have also explored the feasibility of diagnosing heavy metals based on VNIRS. Shi et al. [8] systematically explored the mechanisms of predicted concentrations, acquisition methods, preprocessing technologies, and modeling strategies. Liu et al. [9] investigated the feasibility of estimating heavy metal contaminations in floodplain soils. Similar to the Liu et al. [9], St. Luce et al. [16] explored the feasibility of predicting heavy metal concentrations based on visible near-infrared reflectance spectroscopy. Choe et al. [17] mapped heavy metal pollution in stream sediments by combining geochemistry, field spectroscopy, and hyperspectral remote sensing. Wang et al. [18] used VNIRS to predict low Pb concentrations and further investigated the predictive mechanism of heavy metal concentrations. Liu et al. [19] used VNIRS to try to monitor stress levels of rice with heavy metal pollution in a rice canopy, and recommended a fractal dimension of reflectance with a wavelet transform of 480–850 nm as a comprehensive indicator. Chen et al. [10] performed VINRS of 100 samples to rapidly identify the pollution risk of cadmium and identification of pollution hotspots was achieved by interpolating the predicted values. Shi et al. [11] even considered improving the diagnostic and predicted accuracies using combined VNIRS of rice plants and their soil.

However, these above studies only used a single spectral resolution with 1 nm for heavy metal pollution diagnosing, thus there was a lack of the exploration of the diagnostic ability with the different resolutions. The exploration does help to find an optimal spectral resolution for heavy metal pollution diagnosing, especially for cross contamination diagnosing. Some researches [20,21,22] have indicated that the higher spectral resolution not only increases the spectrometer production cost, but also involves diagnostic accuracy due to Hughes phenomenon [23]. So, it is necessary to explore the optimal spectral resolution for heavy metal cross contamination diagnosing.

Given the importance of optimal spectral resolution, this study aimed to propose a method to determinate the optimal spectral resolution for heavy metal cross contamination diagnosing in agricultural soils. To achieve this goal, the specific objectives were: (1) to design a cross contamination experiment with Cd and Pb with four different pollution concentrations; (2) acquire the hyperspectral reflectance of each sample at spectral resolutions; (3) select the number and combination of feature bands for subsequent diagnosis based on two-way analysis of variance (ANOVA2) and random forest (RF); and (4) determine the optimal spectral resolution for Cd-Pb pollution diagnosing based on diagnostic accuracies of SVM. The result of this study is expected to give a suggestion for sensors’ spectral resolution selection in heavy metal cross contamination diagnosing by using plant reflectance spectroscopy.

## 2. Materials and Methods

### 2.1. Materials

Rice, the dominant staple food in China [18], was used as an indicator for cross contamination diagnosing. Pb was the most seriously polluted source for rice in some southern provinces of China, followed by Cd [24], so Cd and Pb were selected for the pollution sources for cross contamination in this experiment. As opposed to soil cultures, water was used to plant rice to ensure the uniformity of the contaminative concentration. To avoid the influence of environmental factors, the containers were previously painted black [25]. Four different contaminative concentrations of Cd pollution, 0 mg/L, 2 mg/L, 5 mg/L, and 8 mg/L, were used, while the concentrations of Pb pollution were 0 mg/L, 50 mg/L, 100 mg/L, and 500 mg/L. Then, each Cd contaminative concentration as combined with that of Pb. Including the normal group, there were totally 16 groups in the experiment. The details of pollution pretreatments of the different groups are displayed in Table 1. In the paper, ZCd/ZPb, LCd/LPb, MCd/MPb, and HCd/HPb represented zero, low-, medium-, and high-level concentrations of Cd/Pb pollution, respectively. The contaminative concentrations were determined by early pre-test after referring to the Risk Control Standard for Soil Contamination of Agricultural Land (GB15618-2008) of China [26] and previous published papers [9,10,18,27,28,29,30]. These concentrations were also closed to the controlled value in (GB15618-2008)—3 mg/kg and 700 mg/kg for Cd and Pb respectively in farmland soil [26].

An ASD FieldSpec®3 portable spectrometer (ASD Inc., now PANalytical Company, Boulder, CO, USA) with a spectral range of 350–2500 nm was used to measure the raw spectrum, and this equipment performed data collection with 10 scans per second [31,32]. The spectral measurements were conducted on a cloudless, sunny day between 10:00 am and 2:00 pm. For the measurements of each pollution pretreatment group, a standardized plate with 100% reflectance was used to calibrate the reflectance measurement [33]. To ensure the reliability of the measured spectrum, each sample underwent ten parallel measurements, with the mean value used as the final determination of the hyperspectral information. After screening, the spectral datasets, including six times complete measurements, were selected from multiple datasets for subsequent diagnostic research.

Based on the raw reflectance with a 1 nm spectral resolution, we used average to resample to different spectral resolution, and the subsequent diagnostic accuracies were also based on the spectral average. This study acquired ten kinds of hyperspectral dataset with the different resolution from 1 nm to 10 nm.

### 2.2. Methods

**Figure 1:**
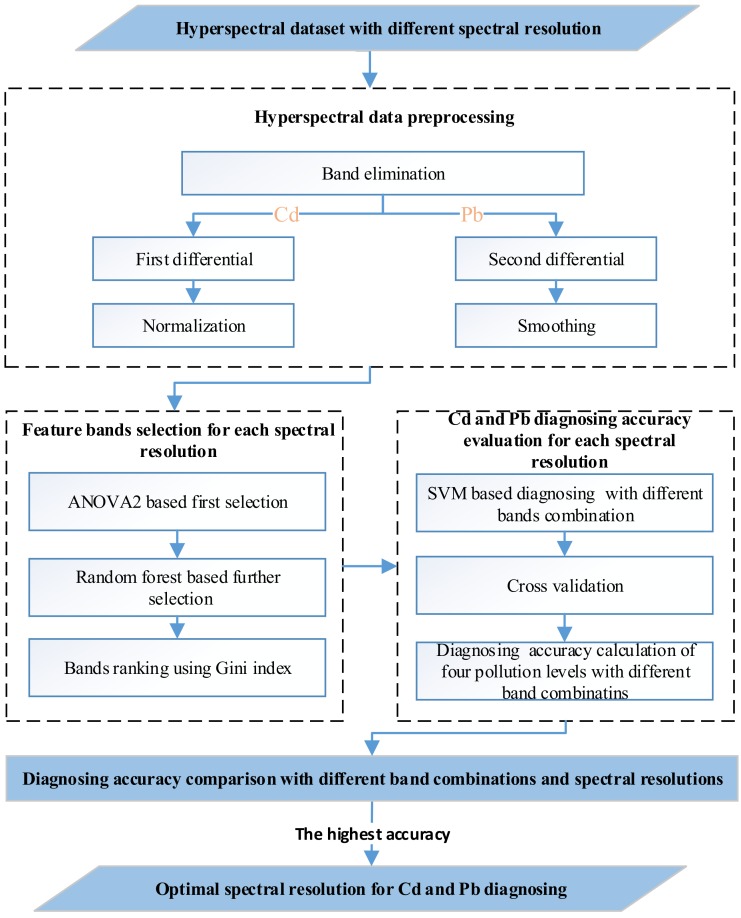
Workflow of the method.

To acquire the optimal spectral resolution for Cd-Pb cross contamination diagnosing, the hyperspectral dataset with different band combinations and spectral resolutions were input the diagnostic model by preprocessing and selection, followed by the accuracy’s comparison. This method mainly included four parts: (1) the hyperspectral preprocessing, (2) feature bands selection, (3) Cd and Pb diagnosing and accuracy evaluation, and (4) diagnosing accuracy comparison with different bands and spectral resolutions. The technological workflow is shown as Figure 1.

#### 2.2.1. Hyperspectral Data Preprocessing

**Figure 2:**
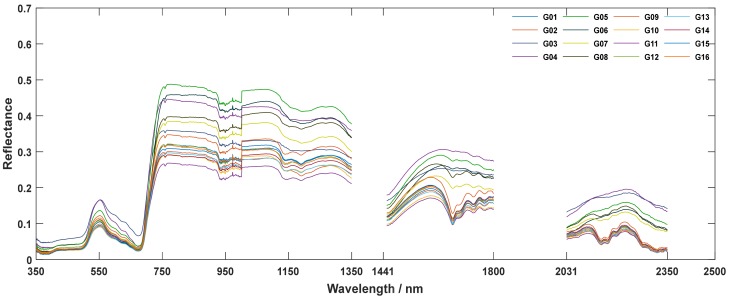
Raw reflectance after removing part of bands for first time measurement.

Due to instrument noise, the spectral bands (1351–1440 nm, 1801–2030 nm, and 2351–2500 nm) were removed to improve the signal-to-noise ratio. The representative curves of remained raw reflectance were shown in Figure 2. To extract differences and eliminate redundancy, the following preprocessing methods were performed for the raw hyperspectral data. The pretreatment of the differentials [34], including the first differential and second differential, reduced the interference of background noise. Savitzky and Golay smoothing [35] eliminated random noise. Normalization or standardization reduced information redundancy and extracted the difference. In practice, the combination of first derivative and normalization and the combination of second derivative and Savitzky and Golay smoothing were used to select the bands that were sensitive to Cd pollution and Pb pollution, respectively.

#### 2.2.2. Feature Bands Selection

The number of input feature bands was one of the key factors for subsequent diagnosis. ANOVA2 and RF were used to determine the number. The three main steps of this analysis were as follows.

ANOVA2 was a statistical analysis method that could be used to analyze whether different levels of two pollutions had a significant impact on the polluted results and distinguished whether there was an interaction between the two pollutions based on the difference significances. Due to the uncertain inducement for interactions effects, ANOVA2 preferentially removed bands that were sensitive to interaction effects, followed by the feature bands first time selection. These feature bands that were sensitive to each of Cd and Pb pollutions were selected with less significance than preset value (0.05).

RF was used to rank the feature bands based on the Gini index and further reduced to ten bands if the bands were more than ten, or all bands were reserved when the aforementioned bands did not reach ten. The Gini index was used as an evaluation indicator to measure the contribution rate of each feature band. The formula for Gini index displayed as following:

(1) $$GI_{m}=\;\sum_{k=1}^{\left|K\right|}\sum_{k^{,}\neq k}p_{mk}p_{mk^{,}}=1-\;\sum_{k=1}^{\left|K\right|}p_{mk}^{2}$$

where *K* is the number of the pollution concentration, and its value is four in the study, $p_{mk}\;$ represents the proportion of the pollution concentration K in the node m. In this experiment, the 0.75 dataset was randomly selected as training data, and the remainder was used for validation datasets. Random forest runs hundreds of times to eliminate the randomness of feature band selection. In each operation, the band with the highest Gini index was reserved, and the feature band ranked on the basis of their frequencies that Gini index was top one. Through testing, we found the feature band importance of each band was stable when the program ran 1000 times repeatedly.

RF ranked the order of the bands based on the Gini index. However, RF did not determine the number of band combinations, an important factor for determining subsequent diagnostic accuracy. The overall diagnostic results of four different contaminative concentrations took the four overall accuracies into consideration, resulting in a relatively sound value, so the overall diagnostic results were used to determine the number of feature bands for each spectral resolution. For each spectral resolution, the diagnostic results were achieved by subsequent SVM model and cross validation with increasing the number of input bands.

#### 2.2.3. Cd and Pb Diagnosing and Accuracy Evaluation

SVM was used as diagnostic models to distinguish the polluted categories and levels. The SVM converted linearly indivisible low-dimensional data into high-dimensional data, making it linearly separable by finding the optimal hyperplane, which was suitable for diagnosing and classifying the study with small samples. All of the programs ran in the Matlab 2015a platform. Through testing, the default settings for the SVM model with the linear kernel function reached the relatively optimal setting values. For any spectral resolution, the SVM model run repeatedly to diagnose the special categories and levels with the increase of the number of input bands from one to ten.

The leave-one-out cross-validation (LOOCV) were used to evaluate the diagnostic model performance. Different from dividing the training dataset and the verification dataset proportionally, the LOOCV method made full use of all hyperspectral data, which eliminated the randomness of diagnosing small data samples. Based on the preset classification label, one data was reserved for verification dataset each time, and the rest were used as training dataset. Finally, the average value of all verification results was used as the accuracy of the diagnostic model.

#### 2.2.4. Diagnosing Accuracy Comparison with Different Bands and Spectral Resolutions

The part aimed at selecting a spectral resolution that was applicable to diagnose a kind of Cd and Pb pollution no matter how the levels changed. The average accuracy of different levels was used as an indicator to determine the optimal bands and the optimal resolution. The highest average accuracy firstly determined the optimal bands combination under a single spectral resolution, and then determined the optimal spectral resolution. The optimal bands and the optimal resolution were determined

(2) $$\mathrm{The}\;\mathrm{optimal}\;\mathrm{bands}\;=\;\left\{\begin{array}{ll} the\;\mathrm{highest}\;\mathrm{AV} & \mathrm{only}\;\mathrm{top}\;\mathrm{value}\\ the\;\mathrm{highest}\;\mathrm{AV}\;\mathrm{with}\;\mathrm{less}\;\mathrm{band}\;\mathrm{number} & \mathrm{two}\;\mathrm{or}\;\mathrm{more}\;\mathrm{top}\;\mathrm{value}\; \end{array}\right.$$

(3) $$\mathrm{The}\;\mathrm{optimal}\;\mathrm{resolution}\;=\;\left\{\begin{array}{ll} the\;\mathrm{highest}\;\mathrm{AV} & \mathrm{only}\;\mathrm{top}\;\mathrm{value}\\ the\;\mathrm{highest}\;\mathrm{AV}\;\mathrm{with}\;\mathrm{less}\;\mathrm{coarser}\;\mathrm{resolution} & \mathrm{two}\;\mathrm{or}\;\mathrm{more}\;\mathrm{top}\;\mathrm{value}\; \end{array}\right.$$

with average accuracy (AV) of different pollution levels.

## 3. Results

### 3.1. Results of Feature Bands Selection

The feature bands were selected by ANOVA2, followed by the random forest algorithm. Before preprocessing, there were 1660 bands. ANOVA2 greatly reduced the dimensions of hyperspectral data by an order of magnitude. The number of sensitive bands of Cd was greater than that of Pb with the exception at the 2 nm resolution. 

**Figure 3:**
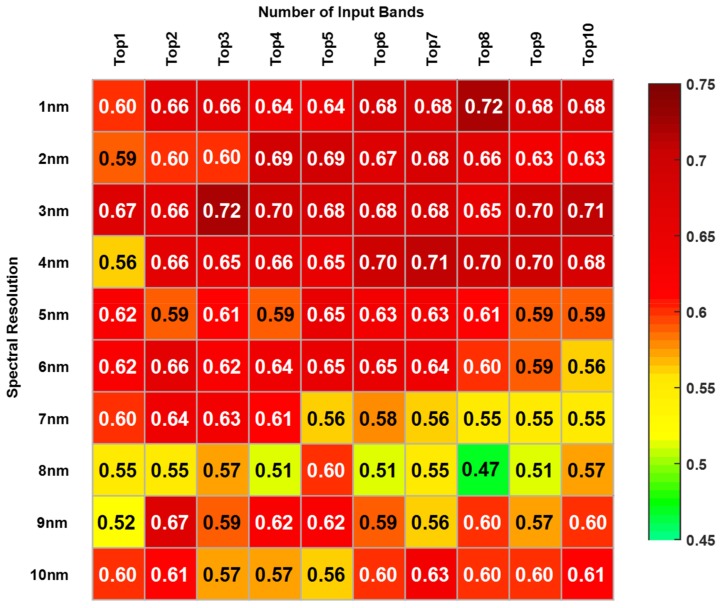
Diagnostic accuracies of Cd pollution diagnosing for band combination selecting.

**Figure 4:**
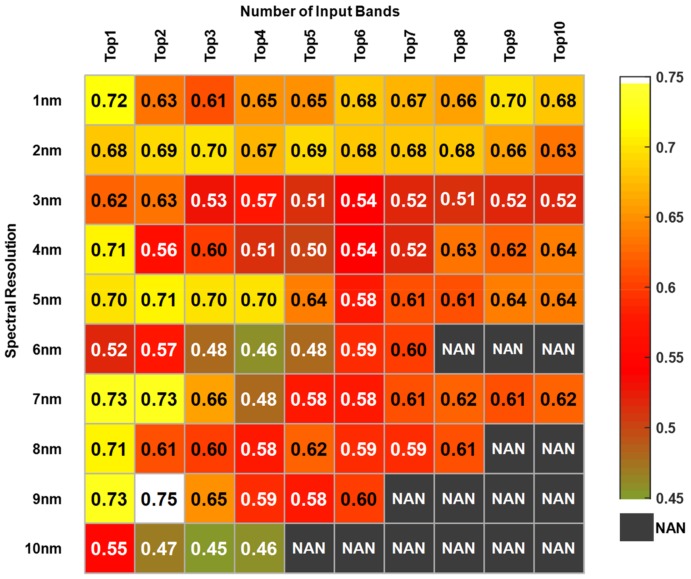
Diagnostic accuracies of Pb pollution diagnosing for band combination selecting.

Figure 3 and Figure 4 showed the changes of the highest accuracies with the increase the number of input bands from one to ten. The x-axis represented the number of input bands, and the y-axis represented the spectral resolution.

As shown in Figure 3, the average accuracies for Cd pollution were distributed in the range from 0.51 to 0.72 except the 0.47 in 8 nm resolution. As the input band changed, the relative highest accuracies of each resolution ranged from 0.60 to 0.72, with a maximum of 0.72 at a 1 nm spectral resolution with eight input bands and 3 nm spectral resolution with three input bands. As the resolution changed, the maximum value of each resolution tended to decrease overall. There was a stable value at 3 nm, and all accuracies were not less 0.65, no matter how the number of input bands changed.

The average accuracies for Pb pollution ranged from 0.45 to 0.75, but nearly half of the highest accuracies were above 0.60, as shown in Figure 4. The accuracies at 1 nm and 2 nm had a distinct advantage, displaying a saffron yellow color. The accuracies at 3 nm were generally low, and the lowest at 10 nm. The highest accuracy of ten resolutions was 0.75 at 9 nm with two input bands. Overall, the resolution of 1 nm and 2 nm stayed relatively stable with accuracies of more than 0.60. The ‘NaN’ represented that there were no feature bands.

By analyzing the diagnostic accuracies with the different band combinations and different resolutions, the optimal bands of each spectral resolution were determined by the highest accuracy. The details of the band combination selection for each spectral resolution were pretend in Table 2. Primitive bands represented the number of the initial bands. ANOVA2 and RF were used to select the band combinations, successively.

After RF selection, the number of band combinations was not more than five for most spectral resolutions. The number of sensitive feature bands of Pb pollution were generally less than that of the Cd bands. For Cd pollution, the number of band combinations did not exceed five in six different spectral resolutions. For Pb pollution, there was only one feature band in five different spectral resolutions.

### 3.2. Diagnostic Accuracies of Different Spectral Resolution for Different Levels

For each resolution, the diagnostic accuracies of each pollution level also depended on the highest accuracy. After diagnosing with SVM and LOOCV validating, followed by band combination selecting, the diagnostic accuracies of different pollution levels were obtained, as shown in Table 3. 

The results indicated that it was not uniform for the optimal diagnostic value of different pollution categories and levels. The highest diagnostic accuracies of the zero, low, medium, and high levels of Cd pollution were 0.75, 0.74, 0.74, and 0.75 at 1 nm, 10 nm, 4 nm, and 3 nm, respectively, while the highest diagnostic accuracies for Pb were 0.85, 0.73, 0.75, and 0.79 at 9 nm, 7 nm, 9 nm, and 6 nm, respectively. The highest diagnostic accuracies were above 0.70 no matter what categories and what levels the rice subject. Among all the highest diagnostic accuracies, the diagnosis of the zero-concentration Pb reached the optimal diagnosis with the accuracy of 0.85.

For the Cd pollution of the zero concentration, the accuracies were not less 0.70 before the spectral resolution increased to the 5 nm. When the spectral resolution expanded 5 nm or more, all accuracies were less than 0.65 except 0.69 at 7 nm. For the low concentration diagnosing, the highest diagnostic accuracy was 0.74 at 10 nm resolution. In addition, the accuracies were more than 0.65 from 1 nm to 4 nm. For the spectral resolution in the range from 1 nm to 7 nm, the diagnostic accuracies of the medium concentration were above 0.65, and the highest diagnostic accuracy was 0.74 at 4 nm. Similar to the diagnosis of the zero concentration, the highest diagnostic accuracy of high concentration was also 0.75, but the spectral resolution was different. Besides the highest diagnostic accuracy at 3 nm, there were five resolutions with an accuracy of not less than 0.7. For the high concentration diagnosis, the accuracies were not less than 0.60 except the poorest value 0.47 at 10 nm. The highest diagnostic accuracy was 0.79 at 6 nm, and the accuracies exceeded 0.65 in the range from 4 nm to 9 nm. 

For Pb pollution, the highest diagnostic accuracy of zero concentration reached 0.85, which was the highest value in all diagnosis of different concentration, and there were two spectral resolutions, 3 nm and 9 nm, that all reached the highest diagnostic accuracy. Except for the spatial resolution of 1 nm and 3 nm, the rest of the accuracies exceeded 0.75. The accuracy at 1nm was the lowest, but it was still close to 0.70. For the low concentration diagnosing, the accuracy fluctuated greatly, and the resolution at 2 nm and 5 nm did not exceed 0.45. The highest diagnostic accuracy was 0.73 at 7 nm, while the minimum was only 0.31 at 3 nm, and the difference was more than 0.4. Similar to the diagnosis of the low concentration, there were two resolutions that their accuracies were less than 0.45, with the worst accuracy being only 0.36 at 10 nm. The highest diagnostic accuracy of medium concentration was 0.75 at 9 nm.

## 4. Discussion

### 4.1. Suitable Wavelengths Analysis for Cd-Pb Pollution Diagnosing

The optimal spectral resolution affects the production cost of the sensor, and the wavelengths coverage is related to the fabrication and spectral acquisition of sensor. So, we investigated the wavelength coverage of the optimal bands for each spectral resolution.

**Figure 5:**
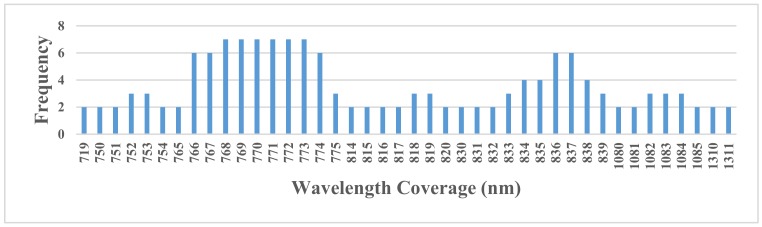
Frequencies of the input bands for the primitive wavelength for Cd pollution.

**Figure 6:**
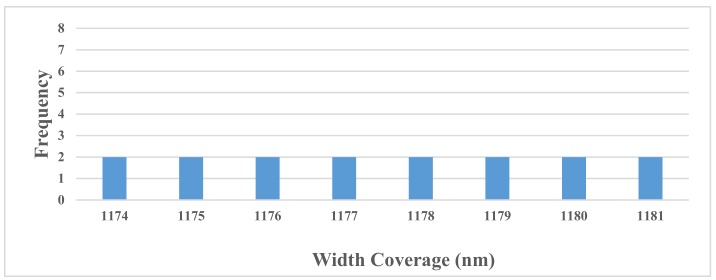
Frequency of the input bands of the primitive wavelength for Pb pollution.

Table 4 and Table 5 show the wavelengths of the input bands from 1 nm to 10 nm for single Cd and Pb diagnosing, and Figure 5 and Figure 6 show the corresponding frequencies of the chosen wavelengths in all ten spectral resolutions. Due to an excess of bands, the figures only show the bands with the frequencies greater than one.

As shown in Figure 5 and Figure 6, there were forty-one bands of primitive reflectance to choose for the Cd pollution diagnosing, while there were only eight bands for Pb pollution. For Cd pollution diagnosing, the highest frequency was seven, including six primitive bands from 768 nm to 773 nm, which were located in the near-visible wavelength coverage. Compared with the frequency of the feature bands diagnosing Cd pollution, the distribution position of the input feature bands with the highest frequency were relatively scattered, and the highest frequency was only two, including eight primitive reflectance bands from 1174 nm to 1181 nm. Thus, subsequent research may consider focusing on 768–773 nm and 1174–1181 nm for Cd-Pb cross contamination diagnosing.

### 4.2. Optimal Spectral Resolution Analysis

**Figure 7:**
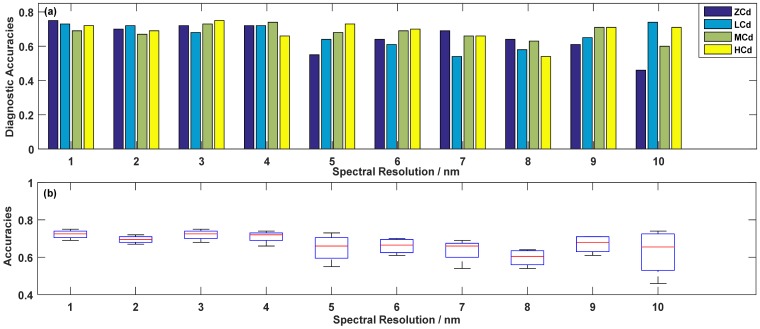
Optimal accuracies (**a**) and corresponding boxplots (**b**) for diagnosing Cd pollution in different resolutions.

**Figure 8:**
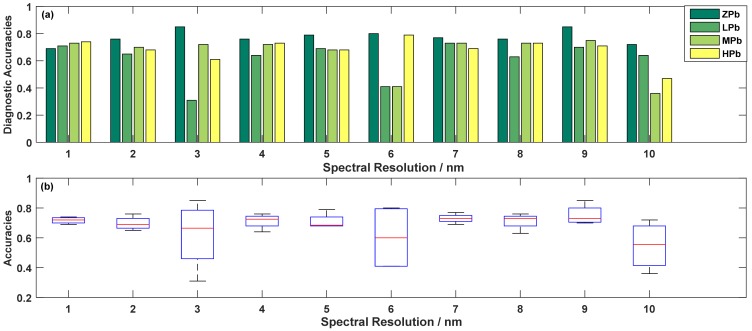
Optimal accuracies (**a**) and corresponding boxplots (**b**) for diagnosing Pb pollution in different resolutions.

The average accuracy of the four levels was a key indicator of diagnosing ability, so the spectral resolution, depending on the highest accuracy with different spectral resolution, could be used as a reference for subsequent Cd-Pb diagnosis and even spectrometer production. Figure 7 and Figure 8 showed the changes of the diagnostic accuracies when only considering a single heavy metal diagnostic results, and Table 6 displayed the highest accuracies of Cd-Pb comprehensive diagnosis after selecting band combination. 

As shown in Figure 7a, for diagnosed Cd pollution, the histograms of ZCd showed a downward trend as a whole, but there was a small ridge at the 4 nm resolution. The low concentration diagnostic histograms showed a trend of rising, falling, and rising again, and the accuracy at 10 nm was peak value. The diagnostic accuracies of the medium concentration slightly fluctuated, between 0.6 and 0.7, and the maximum value appeared at 4 nm. Similar to the diagnosis of the zero concentration, the diagnostic accuracies of the high concentration were generally decreasing, but there was a peak at 3 nm. 

The boxplots in Figure 7b showed the overall diagnostic level for four different Cd contaminative concentrations. There are not outliers for diagnostic accuracies. The resolutions of 1 nm and 3 nm had a clear advantage with the overall better diagnostic level. The detailed accuracies at 1 nm were 0.75, 0.73, 0.69, and 0.72 for zero, low, medium, and high concentration, respectively, while the accuracies at 3 nm were 0.72, 0.68, 0.73, and 0.75, respectively. Taking the sensors production cost into consideration, the 3 nm spectral resolution was a more appropriate spectral resolution for identifying whether the rice was exposed to any concentration of Cd pollution.

As shown in Figure 8a, for Pb pollution under a single level, the highest accuracies were generally not at 1 nm. For the zero-concentration diagnosing, the accuracies were flat between 4 nm and 8 nm, and the maximum value was 0.85 at 3 nm and 9 nm. Due to the larger spectral resolution, the 9 nm was more suitable for diagnosing ZPb concentration. In the low-concentrations histograms, there was a minimum value, which indicated that it was not an appropriate resolution for 2 nm to diagnose the low-concentration Pb pollution. The medium-concentration histograms showed a stable trend in the range from 1 nm to 4 nm, and then fluctuated greatly with the increase of spectral resolution. If only one pollution level was considered, the 7 nm was the most suitable spectral resolution for low-level Pb pollution diagnosing, as 9 nm was suitable for medium-level diagnosis. For diagnosis of the high level, there was an overall trend of falling, rising and falling again, and the accuracies reached the peak value at 6 nm. In addition, the overall accuracy optimization might result in the poor diagnostic accuracy of a certain pollution level diagnosis. There were some lower diagnostic accuracies, such as the LPb diagnosis at 3 nm and the MPb diagnosis at 6 nm, which indicated that it was not appropriate for these spectral resolutions to distinguish the LPb and MPb, respectively.

It was showed for the overall diagnostic level of Pb contamination in Figure 8b. There were four spectral resolutions that the overall diagnostic levels were better than others, including 1 nm, 5 nm, 7 nm and 9 nm. The specific accuracies at 9 nm were 0.85, 0.70, 0.75, and 0.71 for zero, low, medium, and high concentration, respectively, and the corresponding average accuracy reached 0.75, which was the highest values in all the average accuracies. Thus, 9 nm was a more suitable spectral resolution for distinguishing whether rice is exposed to any concentrations of Pb pollution. 

To find a universal spectral resolution for Cd-Pb cross contamination diagnosing, the comprehensive accuracies of different Cd-Pb levels should be considered. Table 6 displayed the average accuracies of Cd-Pb comprehensive diagnosis after selecting band combination.

Except 0.57 at 10 nm, all average accuracies overpassed 0.60. All AV values exceeded 0.65 before the spectral resolution exceeded 5 nm. The average accuracy reached 0.71 at 9 nm, which was also the highest value in all average accuracies. To further confirm the reliability of the highest accuracy, we also calculated the standard deviation, the recall rate, range (xmax–xmin), and variable coefficient of diagnostic accuracies at different resolutions. 

The higher value of recall ratio represented the stronger diagnostic ability for pre-diagnostic concentrations, while the smaller value of standard deviation (SD), ranges, and variable coefficient represented the better stability in different concentrations. The calculated results showed that the diagnostic accuracy at 9 nm had a relatively small standard deviation of 0.07 and a relatively high recall rate of 0.76. In addition, the range and variable coefficient in 9 nm also kept a more stable condition relatively with a value of 0.24 and 0.09 respectively. Although there was a better stability in the resolution of 2 nm, the resolution of 9 nm was higher in AV and recall ration with a good stability. Therefore, the 9 nm was optimal spectral resolution for Cd-Pb cross contamination diagnosing. 

In this study, the diagnostic accuracies by high-resolution measurement were not always more accurate than low-resolutions ones, for example, the diagnostic accuracies of Cd contamination at 8 nm did not outperform the 9 nm ones. The effect might be related to the Hughes phenomenon [23] and the collinearity between hyperspectral bands [20,21]. On the one hand, the spectral averaging eliminated random errors, caused by the working state of the machine in the process of spectral acquisition. On the other hand, some adjacent collinear bands with high sensitivity may be selected preferentially in the process of selecting feature bands, which may cause the loss of spectral information [22,36,37]. As a result, higher accuracies might be obtained in low resolution. In addition, Marceau’s research results [38] showed that the spectral variability within the category was also a cause.

Based on the above results and discussions, we propose that 9 nm is the optimal spectral resolution to sensor production for Cd-Pb cross contamination diagnosing simultaneously in rice, and give a suggestion that spectral resolutions of 3 nm and 9 nm should be the optimal resolutions for diagnosing the single Cd and Pb pollution. The sampling interval of 1.4 nm, like ASD FieldSpec®3, may be appropriate due to the closed relationship between the sensitive bands’ wavelength coverage and short-wave infrared range. Using the optimal spectral resolution may improve diagnostic stability and reduce the cost of the instrument.

## 5. Conclusions

This paper proposed an optimal spectral resolution for diagnosing the categories and levels of Cd-Pb cross contamination in rice based on the hyperspectral dataset. ANOVA2 and RF were used to select the feature bands, followed by SVM and cross validation to get diagnostic accuracies. By analyzing diagnostic results of different band combinations and different spectral resolution, the band combinations and the optimal spectral resolutions, ranging from 1 nm to 10 nm, were chosen based on the highest accuracy. The results indicated that: (1) the hyperspectral technology was a promising method for diagnosing heavy metal cross contamination of Cd and Pb. For each spectral resolution, no matter what categories and levels of Cd and Pb rice suffered, the first-rank average diagnostic accuracies were above 0.6, except for Pb pollution diagnosing with the 10 nm resolution. (2) Wavelengths of 768–773 nm and 1174–1181 nm might be worth exploring to find a suitable index for distinguishing the Cd and Pb pollution. (3) The 9 nm was the optimal spectral resolution to instruct the sensor production for Cd-Pb cross contamination diagnosing.

This experiment only investigated the optimal spectral resolution diagnosing the Cd-Pb cross contamination. It is worthy of exploring the feasibility of the proposed method to determine the optimal spectral resolution of other heavy metals cross contamination in the future research. This future exploration may find a universal resolution diagnosing heavy-metal pollutions, which can help expand the diagnostic scopes of the sensor. 

## Figures and Tables

**Table 1:** Pollution pretreatments of different groups.

Group Name	Pollution Pretreatment	Group Name	Pollution Pretreatment
G01	ZCd-ZPb	G09	LCd-MPb
G02	LCd-ZPb	G10	LCd-HPb
G03	MCd-ZPb	G11	MCd-LPb
G04	HCd-ZPb	G12	MCd-MPb
G05	ZCd-LPb	G13	MCd-HPb
G06	ZCd-MPb	G14	HCd-LPb
G07	ZCd-HPb	G15	HCd-MPb
G08	LCd-LPb	G16	HCd-HPb

**Table 2:** Details of the band number at each spectral resolution.

Spectral Resolution	Primitive Bands	Cd		Pb	
		Bands after ANOVA2	Input Bands after RF	Bands after ANOVA2	Input Bands after RF
1 nm	1660	48	8	50	1
2 nm	830	34	4	31	3
3 nm	552	26	3	20	2
4 nm	415	28	7	11	1
5 nm	332	23	5	14	2
6 nm	275	21	2	7	7
7 nm	235	19	2	14	1
8 nm	207	17	5	8	1
9 nm	183	19	2	6	2
10 nm	166	12	7	4	1

ANOVA2 = two-way analysis of variance; and RF = random forest.

**Table 3:** Details of the diagnostic accuracies with different spectral resolution.

	ZCd	LCd	MCd	HCd	ZPb	LPb	MPb	HPb
1 nm	**0.75**	0.73	0.69	0.72	0.69	0.71	0.73	0.74
2 nm	0.70	0.72	0.67	0.69	0.76	0.65	0.70	0.68
3 nm	0.72	0.68	0.73	**0.75**	**0.85**	0.31	0.72	0.61
4 nm	0.72	0.72	**0.74**	0.66	0.76	0.64	0.72	0.73
5 nm	0.55	0.64	0.68	0.73	0.79	0.69	0.68	0.68
6 nm	0.64	0.61	0.69	0.70	0.80	0.41	0.41	**0.79**
7 nm	0.69	0.54	0.66	0.66	0.77	**0.73**	0.73	0.69
8 nm	0.64	0.58	0.63	0.54	0.76	0.63	0.73	0.73
9 nm	0.61	0.65	0.71	0.71	**0.85**	0.70	**0.75**	0.71
10 nm	0.46	**0.74**	0.60	0.71	0.72	0.64	0.36	0.47

**Table 4:** Spectral location of the Cd pollution feature bands for any resolution.

Spectral Resolution	Band Width
1 nm	734 nm, 754–755 nm, 768–769 nm, 776 nm, 1237 nm, 1309 nm, 1831 nm
2 nm	766–773 nm, 1310–1311 nm
3 nm	719–721 nm, 752–754 nm, 767–775 nm, 818–820 nm, 836–838 nm, 1214–1216 nm, 1310–1312 nm
4 nm	382–385 nm, 750–753 nm, 766–773 nm, 834–837 nm, 1082–1085 nm, 1298–1301 nm
5 nm	765–774 nm, 785–789 nm, 1015–1019 nm, 1080–1084 nm
6 nm	764–775 nm
7 nm	770–776 nm, 833–839 nm
8 nm	766–773 nm, 814–821 nm, 830–837 nm, 1078–1085 nm, 1222–1229 nm
9 nm	746–754 nm, 836–844 nm
10 nm	710–719 nm, 810–819 nm, 830–839 nm, 1020–1029 nm, 1310–1319 nm, 1340–1349 nm

**Table 5:** Spectral location of the Pb pollution feature bands for any resolution.

Spectral Resolution	Band Width
1 nm	761 nm
2 nm	708–709 nm, 762–763 nm
3 nm	638–640 nm, 884–886 nm
4 nm	1174–1177 nm
5 nm	765–769 nm, 1891–1895 nm
6 nm	392–397 nm, 467–481 nm, 518–529 nm, 572–577 nm, 614–619 nm, 1394–1399 nm
7 nm	1771–1777 nm
8 nm	1174–1181 nm
9 nm	1178–1186 nm, 1870–1878 nm
10 nm	920–929 nm

**Table 6:** Statistical results of Cd-Pb comprehensive diagnosis after selecting band combination.

Spectral Resolution	1 nm	2 nm	3 nm	4 nm	5 nm	6 nm	7 nm	8 nm	9 nm	10 nm
AV	0.69	0.69	0.65	0.66	0.66	0.62	0.68	0.63	**0.71**	0.57
Standard Deviation	0.11	**0.03**	0.1	0.12	0.07	0.14	0.06	0.19	**0.07**	0.12
Recall Ratio	**0.83**	0.75	0.69	0.59	0.79	0.61	0.75	0.80	**0.76**	0.61
Range	0.40	**0.13**	0.35	0.43	0.23	0.41	0.23	0.55	**0.24**	0.35
Variable Coefficient	0.16	**0.05**	0.16	0.18	0.10	0.22	0.09	0.30	**0.09**	0.21
